# Supplementary material for: Patterns of diabetes mellitus by age, sex, and province among Iranian Hajj pilgrims and health care delivery during 2012–2022: A retrospective study of 469,581 participants
Source: PLoS One. 2024 Oct 8;19(10):e0311399. doi: 10.1371/journal.pone.0311399 (PMC11460693; doi:10.1371/journal.pone.0311399)
Supplement: Attachment — Submitted filename: PONE-D-24-05546_reviewer comments.pdf [file pone.0311399.s003.pdf]

PLOS ONE

Patterns of Diabetes Mellitus by Age, Sex, and Province among Iranian Hajj Pilgrims and Health Care Delivery during 2012-2022: A Retrospective Study of 469,581 Participants

--Manuscript Draft--

**Manuscript Number:**	PONE-D-24-05546
**Article Type:**	Research Article
**Full Title:**	Patterns of Diabetes Mellitus by Age, Sex, and Province among Iranian Hajj Pilgrims and Health Care Delivery during 2012-2022: A Retrospective Study of 469,581 Participants
**Short Title:**	Patterns of Diabetes Mellitus by Age, Sex, and Province among Iranian Hajj Pilgrims during 2012-2022
**Corresponding Author:**	Samad Azari<br>Iran University of Medical Sciences<br>Tehran, Tehran IRAN, ISLAMIC REPUBLIC OF
**Keywords:**	Diabetes Mellitus; Hajj; Prevalence; Iran
**Abstract:**	Objective<br><br>Hajj is among the oldest religious ceremonies in the world, there is a limited study that evaluates the epidemiological pattern of diabetes and the medical care required and provided to pilgrims. The present study assessed the prevalence and pattern of diabetes mellitus in Iranian pilgrims in 2012-22.<br><br>Method<br><br>All demographic information, risk factors, and the prevalence of DM were extracted from the database and medical records of the Hajj Pilgrimage Medical Centre, Iranian Red Crescent Society through file reading. Also, to investigate the effect of the risk factors considered in the study, the multiple logistic regression model was used.<br><br>Results<br><br>The present study included data from 469,581 hajj pilgrims. Most pilgrims were in the age group of 45 to 70 years (73.25%). The prevalence of diabetes in patients over 70 years old was the highest (16.73%). The prevalence of DM was estimated at 14.64% in women and 12.51% in men. The lowest DM prevalence was in Lorestan (7.81%), North Khorasan (9.07%), Sistan and Baluchistan (9.29%), and Hamedan (9.41), respectively. And highest prevalence rate was in Khuzestan (20.12%), Yazd (19.14), and Mazandaran (17.55), respectively. Our analysis reveals that, for instance, with each yearly increase in the age of the pilgrims (assuming other variables remain constant), the odds of having DM increase by 0.04. For the gender, the odds of having DM among women is 0.33 higher than among men, when the other variable is constant.<br><br>Conclusions<br><br>The study results show a significant difference in the prevalence of diabetes in age, gender, and distribution in different provinces. Therefore, appropriate screening, diagnosis, and management by primary care physicians are necessary to prevent adverse health outcomes and reduce the economic burden of mortality and morbidity.
**Order of Authors:**	Pirhossein Kolivand, Ph.D
	Peyman Saberian, MD
	Hossein Saffari, MD
	Taher Doroudi, MD

Powered by Editorial Manager® and ProduXion Manager® from Aries Systems Corporation

	Ali Marashi, MD
	Masoud Behzadifar, Ph.D
	Fereshteh Karimi, Ph.D
	Soheila Rajaei
	Behzad Raei, Ph.D
	Seyed Jafar Ehsanzadeh
	Arash Parvari, Ph.D
	Samad Azari, Ph.D
**Additional Information:**	
**Question**	**Response**
**Financial Disclosure**<br><br>Enter a financial disclosure statement that describes the sources of funding for the work included in this submission. Review the submission guidelines for detailed requirements. View published research articles from *PLOS ONE* for specific examples.<br><br>This statement is required for submission and **will appear in the published article** if the submission is accepted. Please make sure it is accurate.<br><br>**Funded studies**<br>Enter a statement with the following details:<br>• Initials of the authors who received each award<br>• Grant numbers awarded to each author<br>• The full name of each funder<br>• URL of each funder website<br>• Did the sponsors or funders play any role in the study design, data collection and analysis, decision to publish, or preparation of the manuscript?<br><br>Did you receive funding for this work?	The author(s) received no specific funding for this work.
**Competing Interests**<br><br>Use the instructions below to enter a competing interest statement for this submission. On behalf of all authors, disclose any competing interests that could be perceived to bias this	The authors have declared that no competing interests exist.

work—acknowledging all financial support and any other relevant financial or non-financial competing interests.<br><br>This statement is required for submission and **will appear in the published article** if the submission is accepted. Please make sure it is accurate and that any funding sources listed in your Funding Information later in the submission form are also declared in your Financial Disclosure statement.<br><br>View published research articles from *PLOS ONE* for specific examples.<br><br>**NO authors have competing interests**<br><br>Enter: *The authors have declared that no competing interests exist.*<br><br>**Authors with competing interests**<br><br>Enter competing interest details beginning with this statement:<br><br>*I have read the journal's policy and the authors of this manuscript have the following competing interests: [insert competing interests here]*<br><br>* typeset	
**Ethics Statement**<br><br>Enter an ethics statement for this submission. This statement is required if the study involved:<br><br>• Human participants<br>• Human specimens or tissue<br>• Vertebrate animals or cephalopods<br>• Vertebrate embryos or tissues<br>• Field research<br><br>Write "N/A" if the submission does not require an ethics statement.	This study is an extract from the research project with the Code of Ethics IR.RCS.REC.1401.023 from the Iranian Red Crescent Society, which has been conducted at the Research Center for Emergency and Disaster Resilience, Red Crescent Society of the Islamic Republic of Iran, Tehran, Iran. Due to the retrospective nature of the study claims, data were analyzed anonymously written informed consent was waived according to the Research Ethics Committee of the Iranian Red Crescent Society before the start of the study. All stages of the study, including the design, implementation, and reporting, were conducted without any involvement from patients or the public. All methods were carried out according to Helsinki guidelines and regulations.

Powered by Editorial Manager® and ProduXion Manager® from Aries Systems Corporation

General guidance is provided below. Consult the submission guidelines for detailed instructions. **Make sure that all information entered here is included in the Methods section of the manuscript.**<br><br>**Format for specific study types**<br><br>**Human Subject Research (involving human participants and/or tissue)**<br>• Give the name of the institutional review board or ethics committee that approved the study<br>• Include the approval number and/or a statement indicating approval of this research<br>• Indicate the form of consent obtained (written/oral) or the reason that consent was not obtained (e.g. the data were analyzed anonymously)<br><br>**Animal Research (involving vertebrate animals, embryos or tissues)**<br>• Provide the name of the Institutional Animal Care and Use Committee (IACUC) or other relevant ethics board that reviewed the study protocol, and indicate whether they approved this research or granted a formal waiver of ethical approval<br>• Include an approval number if one was obtained<br>• If the study involved *non-human primates*, add *additional details* about animal welfare and steps taken to ameliorate suffering<br>• If anesthesia, euthanasia, or any kind of animal sacrifice is part of the study, include briefly which substances and/or methods were applied<br><br>**Field Research**<br><br>Include the following details if this study involves the collection of plant, animal, or other materials from a natural setting:<br>• Field permit number<br>• Name of the institution or relevant body that granted permission	
**Data Availability**<br><br>Authors are required to make all data underlying the findings described fully	Yes - all data are fully available without restriction

available, without restriction, and from the time of publication. PLOS allows rare exceptions to address legal and ethical concerns. See the PLOS Data Policy and FAQ for detailed information.<br><br>A Data Availability Statement describing where the data can be found is required at submission. Your answers to this question constitute the Data Availability Statement and **will be published in the article**, if accepted.<br><br>**Important:** Stating 'data available on request from the author' is not sufficient. If your data are only available upon request, select 'No' for the first question and explain your exceptional situation in the text box.<br><br>Do the authors confirm that all data underlying the findings described in their manuscript are fully available without restriction?	
**Describe where the data may be found in full sentences. If you are copying our sample text, replace any instances of XXX with the appropriate details.**<br><br>• If the data are **held or will be held in a public repository**, include URLs, accession numbers or DOIs. If this information will only be available after acceptance, indicate this by ticking the box below. For example: *All XXX files are available from the XXX database (accession number(s) XXX, XXX.*).<br>• If the data are all contained **within the manuscript and/or Supporting Information files**, enter the following: *All relevant data are within the manuscript and its Supporting Information files.*<br>• If neither of these applies but you are able to provide **details of access elsewhere**, with or without limitations, please do so. For example:<br><br>*Data cannot be shared publicly because of [XXX]. Data are available from the XXX Institutional Data Access / Ethics*	NA

Committee (contact via XXX) for researchers who meet the criteria for access to confidential data.<br><br>*The data underlying the results presented in the study are available from (include the name of the third party and contact information or URL).*<br>• This text is appropriate if the data are owned by a third party and authors do not have permission to share the data.<br><br>* typeset	
Additional data availability information:	

Powered by Editorial Manager® and ProduXion Manager® from Aries Systems Corporation

Manuscript

Click here to access/download;Manuscript;DM Manuscript Clear.docx

Patterns of Diabetes Mellitus by Age, Sex, and Province among Iranian Hajj Pilgrims and Health Care Delivery during 2012-2022: A Retrospective Study of 469,581 Participants

Pirhossein Kolivand[1†], Peyman Saberian[2†], Hossein Saffari[3], Taher Doroudi[4,5], Ali Marashi[4], Masoud Behzadifar[6], Fereshteh Karimi[7], Soheila Rajaei[7], Behzad Raei[8], Seyed Jafar Ehsanzadeh[9], Arash Parvari[10], Samad Azari[11,7]

1: Department of Health Economics, Faculty of Medicine, Shahed University, Tehran, Iran.

2: Department of Anesthesiology, Imam Khomeini Hospital Complex, Tehran University of Medical Sciences, Tehran, Iran.

3: Hasheminejad Kidney Center (HKC), Iran University of Medical Sciences (IUMS), Tehran, Iran.

4: Iranian Red Crescent Society, Haj Medical Center. Tehran, Iran.

5: Shefa Neuroscience Research Center, Khatam Al anbia Hospital. Tehran, Iran.

6: Social Determinants of Health Research Center, Lorestan University of Medical Sciences, Khorramabad, Iran.

7: Research Center for Emergency and Disaster Resilience, Red Crescent Society of the Islamic Republic of Iran, Tehran, Iran.

8: Razi Educational and Therapeutic Center, Tabriz University of Medical Science, Tabriz, Iran.

9: English Language Department, School of Health Management and Information Sciences, Iran University of Medical Sciences, Tehran, Iran.

10: Department of Epidemiology and Biostatistics, School of Public Health, Tehran University of Medical Sciences, Tehran, Iran.

11: Hospital Management Research Center, Health Management Research Institute, Iran University of Medical Sciences, Tehran, Iran.

† Contributed equally as the first authors

Corresponding author: Samad Azari Email: Samadazari1010@gmail.com

Abstract

Objective: Hajj is among the oldest religious ceremonies in the world, there is a limited study that evaluates the epidemiological pattern of diabetes and the medical care required and provided to pilgrims. The present study assessed the prevalence and pattern of diabetes mellitus in Iranian pilgrims in 2012-22.

Method: All demographic information, risk factors, and the prevalence of DM were extracted from the database and medical records of the Hajj Pilgrimage Medical Centre, Iranian Red Crescent Society through file reading. Also, to investigate the effect of the risk factors considered in the study, the multiple logistic regression model was used.

Results: The present study included data from 469,581 hajj pilgrims. Most pilgrims were in the age group of 45 to 70 years (73.25%). The prevalence of diabetes in patients over 70 years old was the highest (16.73%). The prevalence of DM was estimated at 14.64 % in women and 12.51 % in men. The lowest DM prevalence was in Lorestan (7.81%), North Khorasan (9.07%), Sistan and Baluchistan (9.29%), and Hamedan (9.41), respectively. And highest prevalence rate was in Khuzestan (20.12%), Yazd (19.14), and Mazandaran (17.55), respectively. Our analysis reveals that, for instance, with each yearly increase in the age of the pilgrims (assuming other variables remain constant), the odds of having DM increase by 0.04. For the gender, the odds of having DM among women is 0.33 higher than among men, when the other variable is constant.

Conclusions: The study results show a significant difference in the prevalence of diabetes in age, gender, and distribution in different provinces. Therefore, appropriate screening, diagnosis, and management by primary care physicians are necessary to prevent adverse health outcomes and reduce the economic burden of mortality and morbidity.

Keywords: Diabetes Mellitus, Hajj, Prevalence**,** Iran

Introduction

The Hajj, an annual ceremony, is one of the largest mass gatherings worldwide, attracting over 3.5 million Hajj pilgrims from 200 countries to perform the Hajj rituals in Mecca, the Kingdom of Saudi Arabia (KSA). Successful participation in this ritual requires meticulous preparations undertaken months in advance. The increasing number of pilgrims challenges global and public health security for KSA and countries of various nationalities and races. These challenges encompass housing, food, water, transportation, communication, sanitation, mass-gathering and crowd control, and security, with the density of people reaching up to 7 individuals per square meter (2, 3).

Diabetes is a serious, chronic disease characterized by elevated blood glucose concentrations due to the effects of abnormal β-cell biology on insulin action. Diabetes mellitus (DM) is one of the leading causes of death and disability worldwide, affecting individuals regardless of their country, age group, or sex. In 2017, the expenses incurred by health systems due to diabetes were estimated to be 727 billion. The number of people suffering from diabetes was estimated to be 285 million in 2009, 415 million in 2015, and 529 million (95% uncertainty interval [UI] 500–564) in 2021, indicating a continuous increase in global cases (4, 7, 8). The prevalence of diabetes is high in several countries with a majority Muslim population, such as Pakistan, Indonesia, Egypt, Bangladesh, Iran, and India. All these countries rank among the top ten in the world in terms of diabetes prevalence. Based on a global data analysis, it is estimated that 148 million Muslims are affected by diabetes (9, 10).

Mass-gathering medicine at Hajj is challenged by issues of high morbidity, healthcare accessibility, patient management, and evacuation, especially in emergencies (11, 12). Limited studies have evaluated the epidemiological pattern of diabetes and the medical care required and provided to pilgrims. The present study evaluated the prevalence and pattern of DM in Iranian Hajj pilgrims during the years 2012-22. Given the high population of pilgrims in the study, the results can be used to develop preventive and health protocols. This study will provide a comprehensive overview of diabetes status in pilgrims over ten years.

Methodology

Ethics approval and consent to participate

This study is an extract from the research project with the Code of Ethics IR.RCS.REC.1401.023 from the Iranian Red Crescent Society, which has been conducted at the Research Center for Emergency and Disaster Resilience, Red Crescent Society of the Islamic Republic of Iran, Tehran, Iran. Due to the retrospective nature of the study claims, data were analyzed anonymously, and written informed consent was waived according to the Research Ethics Committee of the Iranian Red Crescent Society before the start of the study. All stages of the study, including the design, implementation, and reporting, were conducted without any involvement from patients or the public. All methods were carried out according to Helsinki guidelines and regulations.

Research Method

The present study was conducted in 2023. In this study, the prevalence of Diabetes Mellitus (DM) was examined by gender, province, and year from 2012 to 2022. All demographic information, risk factors, and the prevalence of diabetes were extracted from the databases of the Hajj and Pilgrimage Medical Center through file reading. Overall, this study investigated the age distribution of pilgrims, the prevalence of Diabetes Mellitus (DM) by age and gender among pilgrims in each age group, and the prevalence of DM among pilgrims by province and year.

Sampling and Inclusion & Exclusion criteria:

No sampling was made in this study, and all Hajj pilgrims during the study period (from 2012 to 2022) were included in the analysis. We also excluded Pilgrims whose information was not fully recorded in the databases. Data were accessed for research purposes on 25.04.2023.

Age Category:

In the present study, pilgrims were categorized into four age groups, under 15 years, 15-44 years, 45-69 years, and over 70-year-old pilgrims, respectively.

Data Analysis and Statistical Tests

The extracted data were entered into a researcher-built form and analyzed using Excel 2019, SPSS 26, Stata 17, and R software version 4.3.1. To investigate the effect of the risk factors considered in the study, such as Fasting Blood Sugar (FBS), age, and sex on the odds of diabetes disease in pilgrims, a multiple logistic regression model was used. Given that the prevalence of DM among pilgrims varies across provinces, and pilgrims in the same province are more similar than those in other provinces, we considered the province as a cluster. We used Robust Standard Error to adjust

the correlation between pilgrims in the same province. Statistical analyses were conducted with a predetermined significance level of 5% ($\alpha = 0.05$).

Results:

Characteristics of the study sample

In total, the present study included data from 469,581 hajj pilgrims, about 50% females and 50% males. Of the total pilgrims, 20.92% were hypertensive, and 13.55% had DM. The highest number of pilgrims, 89,492 individuals, belonged to the year 2019, and 73.25% were in the group aged 45–70 years (Table 1).

Table 1. Characteristics of Iranian hajj pilgrims

**Variables**	N(%)
**Sex**	
Male	234,773 (49.99%)
Female	234,808 (50.01%)
**Age** (years)	
<15	133 (0.02%)
15-44	75,063 (15.98%)
45-69	343,977 (73.25%)
70≤	50,408 (10.75%)
**Diabetes**	
Yes	63,637 (13.55%)
No	405,944 (86.45%)
**Hypertension**	
Yes	98,264 (20.92%)
No	371,317 (79.08%)
**Year**	
2012	61,456 (13.09%)
2013	61,364 (13.07%)
2014	61,853 (13.17%)
2016	71,053 (15.13%)
2017	85,416 (18.19%)

2019	89,492 (19.06%)
2022	38,947 (8.29%)

Prevalence of DM by Sex and Age

According to the findings, the prevalence of DM among pilgrims increases with age, with the highest prevalence of the disease observed in pilgrims aged 70 years and above (Table 2).

Table 2. Prevalence of DM in hajj pilgrims by age

Age (years)	DM, N (%)		Total
	No	Yes	-
<15	133	0(0.0%)	133
15≤age<45	72,761	2,211(2.94%)	74,972
45≤age<70	290,497	53,006(15.43%)	343,503
70≥	41,919	8,420(16.72%)	50,339

Prevalence of DM by province

As indicated by the data in the graph, the prevalence of diabetes among Hajj pilgrims from Lorestan province was the lowest among the 31 provinces. Of the 7110 pilgrims who journeyed to Mecca between 2012 and 2022, only 555 were diagnosed with diabetes, resulting in an estimated disease prevalence of 7.81%. Conversely, Khuzestan province exhibited the highest prevalence of this disease. Out of 24290 pilgrims, 4888 were affected by diabetes, yielding a prevalence rate of 20.12% in this province. These findings highlight the significant regional disparities in diabetes prevalence among Hajj pilgrims.

Table 3. Prevalence of Diabetes by province among Hajj pilgrims

Province	DM		Total
	0	1	
**Chahar Mahaal and Bakhtiari**	2,179 (87.40)	314 (12.60)	2,493 (100)
**Kohgiluyeh and Boyer-Ahmad**	738 (86.11)	119 (13.89)	857 (100)
**Alborz**	6,854 (85.79)	1,135 (14.21)	7,989 (100)
**Ardabil**	3,473 (85.61)	584 (14.39)	4,057 (100)
**Bushehr**	4,661 (86.41)	733 (13.59)	5,394 (100)
**East Azarbayjan**	13,264 (87.00)	1,982 (13.00)	15,246 (100)
**Fars**	19,277	2,875	22,152

Province	DM		Total
	0	1	
	(87.02)	(12.98)	(100)
Gilan	3,139<br>(83.75)	609<br>(16.25)	3,748<br>(100)
Golestan	18,937<br>(87.17)	2,787<br>(12.83)	21,724<br>(100)
Hamadan	11,807<br>(90.59)	1,227<br>(9. 41)	13,034<br>(100)
Hormozgan	4,938<br>(88.27)	656<br>(11.73)	5,594<br>(100)
Ilam	617<br>(86.78)	94<br>(13.22)	711<br>(100)
Esfahan	38,020<br>(86.37)	6000<br>(13.63)	44,020<br>(100)
Kerman	14,930<br>(85.15)	2,604<br>(14.85)	17,534<br>(100)
Kermanshah	4,779<br>(87.69)	671<br>(12.31)	5,450<br>(100)
Khuzestan	19,402<br>(79.88)	4,888<br>(20.12)	24,290<br>(100)
Kurdistan	7,906<br>(87.75)	1,093<br>(12.15)	8,999<br>(100)
Lorestan	6,555<br>(92.19)	555<br>(7.81)	7,110<br>(100)
Markazi	8,159<br>(99.86)	1,080<br>(0.14)	9,239<br>(100)
Mazandaran	17,510<br>(82.45)	3,727<br>(17.55)	21,237<br>(100)
North Khorasan	6,534<br>(90.93)	652<br>(9.07)	7,186<br>(100)
Qazvin	5,448<br>(85.02)	960<br>(14.98)	6,408<br>(100)
Qom	11,710<br>(87.66)	1,649<br>(12.34)	13,359<br>(100)
Razavi Khorasan	49,566<br>(87.07)	8,020<br>(13.93)	57,586<br>(100)
Semnan	5,707<br>(86.82)	866<br>(13.18)	6,573<br>(100)
Sistan and Baluchistan	8,399<br>(90.71)	860<br>(9.29)	9,259<br>(100)
South Khorasan	8,953<br>(87.32)	1,300<br>(12.68)	10,253<br>(100)
Tehran	63,383<br>(87.99)	8,655<br>(12.01)	72,038<br>(100)
West Azarbayjan	17,713<br>(85.90)	2,908<br>(14.10)	20,621<br>(100)
Yazd	13,371<br>(80.86)	3,166<br>(19.14)	16,537<br>(100)
Zanjan	7,381<br>(89.48)	868<br>(10.52)	8,249<br>(100)
Total	405,310<br>(86.43)	63,637<br>(13.57)	468,947<br>(100)

Factors associated with the prevalence of DM

The impact of demographic variables and certain risk factors on diabetes has been evaluated. Given that the presence of diabetes is a dichotomous variable (DM=0 for absence, DM=1 for presence), a logistic regression model was employed. The data exhibited a clustered structure, suggesting that individuals within a province may share similarities in terms of certain environmental and genetic characteristics. As the objective of the study was not to draw inferences about the provinces, robust standard errors (Robust SE) were utilized to adjust for this clustering, thereby facilitating more accurate inferences. This approach ensures a rigorous analysis while accounting for the inherent structure of the data.

Table 4. Results of logistic regression analysis for factors influencing the prevalence of DM

DM	OR	Robust. St.Err.	t-value	p-value	[95% Conf	Interval]	Sig
Age	1.039	.003	13.83	0	1.034	1.045	***
Sex (Women=1)	1.329	.041	9.34	0	1.252	1.411	***
FBS (≥ 126)	29.842	1.407	72.02	0	27.208	32.732	***
Constant	.005	.001	-37.26	0	.004	.006	***

Mean dependent var	0.136	SD dependent var	0.342
Pseudo r-squared	0.303	Number of obs	468947
Chi-square	7623.526	Prob > chi2	0.000
Akaike crit. (AIC)	259495.904	Bayesian crit.	259540.137

**** p<.01, ** p<.05, * p<.1*

The chi-square statistic for the overall model was estimated to be 7623.526, indicating that the fitted model is statistically significant (p-value<0.001). The null hypothesis, which posits that all independent variables in the model do not affect the odds (probability) of having DM, has been rejected. This suggests that at least one of the independent variables in the model significantly influences the odds (probability) of developing DM. The p-value for all independent variables in the model was less than 0.05, implying that all variables significantly affect the odds of developing DM.

Our analysis reveals that, for instance, with each yearly increase in the age of the pilgrims (assuming other variables remain constant), the odds of having DM increase by 0.04. For the gender, the odds of having DM among women is 0.33 higher than among men, when the other variable is constant. Similarly, for the fasting blood sugar variable, the odds of having DM for individuals with a fasting blood sugar level greater or equal to 126 ($FBS \geq 126$) are 29.84 times the odds of having DM for individuals with a fasting blood sugar level less than 126 ($FBS < 126$), when other variables are considered to be constant.

DM Healthcare Delivery and Economic Burden:

DM has been one of the most important risk factors and diseases among Hajj pilgrims. DM can raise morbidity and mortality and lead to an increase in the consumption of medical services and the economic burden of disease (Direct cost and indirect cost).

The trend of providing DM services to Hajj pilgrims was also reported in 2018, 2019, 2022, and 2023. The highest rate of receiving DM services was in 2023 with 37160 and the lowest rate was in 2022 with 2727 cases requiring dialysis treatment. The results of our study show that according to the data from Hajj Pilgrimage Medical Centre, about 10% of the total medical services provided during the 2023 Hajj ceremony were for DM outpatient and inpatient services.

Discussion

The primary objective of this study is to gather demographic data, estimate the prevalence of DM, and identify its influencing factors among Iranian pilgrims from 2012 to 2022. The analysis of DM prevalence in the target population reveals four key findings. The first finding shows that the highest prevalence of DM was observed in the age group of 70 years and above, followed by the group aged 45 to 70 years. A study conducted among individuals aged 35 to 70 years, using data from the Persian cohort study, estimated the age-standardized prevalence of diabetes at 15%, which aligns with the findings of the current research (13). A similar study on the trend of DM prevalence among individuals aged 25 to 64 years reported the prevalence of diabetes as 8.4%, 9%, 11.1%, and 13.2% in 2004, 2007, 2011, and 2016, respectively (14). The discrepancy between these findings and the current analysis could be attributed to the age differences of the study groups. A study conducted in Kerman province revealed that the overall prevalence of DM is 12%,

ranging from 7.1% in the age group of 15-24 years to 18.4% in the age group of 55-64 years (15). The prevalence of DM in phase one was estimated at 10.8% and in phase two at 10.3% (15). These findings underscore the significant impact of age on the prevalence of DM among Iranian Hajj pilgrims.

The second finding of this study reveals that the prevalence of diabetes among women is higher than among men, with 12.51% of male pilgrims and 14.61% of female pilgrims diagnosed with this disease. A similar national-level study aligns with these findings, reporting the prevalence of DM as 13.5% in men, 14.7% in women, and 14.2% in both genders (16). A systematic review, which reported the prevalence of DM in 25 papers, found rates ranging from 1.6% among Omani pilgrims to 40% among Chinese pilgrims. The weighted cumulative prevalence of DM among all pilgrims was estimated at 5%. This review study reported the lowest rate of DM prevalence among Australian and Iranian pilgrims (17). A study on the prevalence of chronic diseases in Iranian pilgrims referred to Iranian clinics located in Iraq found that 13.4% of the referred pilgrims suffer from diabetes, with a higher prevalence in female pilgrims than in males (18). Another study assessing the health status of Iranian pilgrims before departing for Hajj over different years showed an increasing trend of diabetes prevalence from 2.44% in 2004 to 8.31% in 2008 (19). A significant study on French pilgrims revealed that the rate of diabetes in French pilgrims is five times higher than that in the general population, with the prevalence of diabetes, hypertension, and obesity being higher in female pilgrims than in male pilgrims (20). The prevalence of chronic diseases is higher in Arab and Muslim countries than in non-Muslim countries. Furthermore, a large portion of the participants in the Hajj ceremony are over 60 years old (21). A study on chronic diseases of deceased hospitalized individuals in Saudi hospitals during the Hajj ceremony showed that one-third of the deceased individuals suffered from diabetes (22). In 2015, it was estimated that there were 415 million people with diabetes between the ages of 20 to 79 years, 5 million deaths due to diabetes, and three-quarters of those with diabetes lived in low- and middle-income countries. It is predicted that the number of people with diabetes between the ages of 20 to 79 will reach 642 million by the year 2040 (23). A study in Malaysia showed that 26.5% of Hajj pilgrims in this country suffer from hypertension and 15.4% from diabetes (24). The findings of a review of 22 selected studies show that the main health problems of pilgrims during Hajj are pneumonia, influenza, and asthma, and diseases such as stroke and heart attack (16.67%) and diabetes (13.33%)

(25). The study by Khan and colleagues shows that analgesics, antibiotics, antacids, antihistamines, as well as diabetes and blood pressure medications were the most commonly prescribed drugs for Indian pilgrims (11). Evidence from a longitudinal study on South African pilgrims indicates that high blood pressure and mostly type 2 diabetes are the most common diseases in these individuals, with prevalence rates of 22.6% and 13.2% respectively (26). A study on the risk factors for hospitalization and mortality among Indonesian pilgrims shows that diabetes significantly increases the risk of hospitalization in Hajj pilgrims (27).

The third finding of this study pertains to the variations in diabetes prevalence among pilgrims from different provinces. A study indicates that the lowest prevalence of diabetes is in Kermanshah province, while the highest rate is observed in Khuzestan province. This aligns with the findings of the current study, which also reports the highest rate of diabetes prevalence among pilgrims from Khuzestan province (16). However, a recently published study presents different findings, showing that the highest and lowest prevalence of diabetes are respectively in Yazd and Urmia. Another study reports that the highest prevalence of prediabetes was observed in Sari, while the lowest was in the city of Fasa (13). These findings underscore the significant regional disparities in diabetes prevalence among Hajj pilgrims.

The fourth finding of this study pertains to the risk factors and determinants of DM prevalence. The results of the current study indicate that a higher probability of developing DM is associated with increasing age, high fasting blood sugar in pilgrims, and female gender. A study in this field reveals that the prevalence of DM is higher in urban residents compared to rural ones and in individuals with lower education levels (16). According to the findings of an internal study, age and gender are determinants of the 5-year incidence rate of diabetes, such that increasing age, high blood pressure, overweight, and obesity are associated with an increased risk of diabetes (28).

The results of multivariate logistic regression in the study by Johari and colleagues in Fars province (Iran) showed that the prevalence of DM is linked with increasing age, being single, family history of diabetes, abdominal obesity, and hypertension. An inverse statistical relationship was observed between the prevalence of DM and factors such as high triglycerides, living in rural areas, higher education, and employment (29). The significant increase in diabetes has occurred in all countries and both rural and urban areas. Accurate global, regional, and local estimates and predictions of

DM prevalence are essential for planning and monitoring prevention and treatment strategies and assessing progress toward achieving the goals set globally for the control of non-communicable diseases and sustainable development.

In a prospective observational study, 26% of individuals hospitalized in the hospitals of the city of Mecca were diagnosed with diabetes, while previously they were unaware of their diabetes. Upon admission of patients, it was found that approximately 77% of the participants had poor diabetes control and about 55% had received diabetes education sessions before going to Hajj. The study findings showed that approximately 37% of hospitalized individuals sometimes monitor their blood sugar themselves and only 22% knew that self-monitoring of blood sugar during the Hajj ceremony is required (30). These findings underscore the significant impact of demographic and health factors on the prevalence of DM among Hajj pilgrims.

Hajj, one of the five pillars of Islam, is a mandatory religious duty for Muslims that must be performed at least once in their lifetime. Considering the global prevalence of diabetes at 8.8%, and the number of adult Muslims performing Hajj (approximately 2.5 million), it can be estimated that the number of Muslims with diabetes performing Hajj may exceed 220,000 annually. Islamic rulings stipulate that Hajj should not impose undue hardship on Muslims. The Holy Quran specifically exempts those who are physically or financially incapable from performing Hajj if it would result in harmful consequences for the individual. This exemption could apply to individuals with diabetes, given the severe and chronic complications associated with the condition. During Hajj, adherence to dietary guidelines, fluid intake, and mobility is crucial. Poor dietary control in pilgrims with diabetes can lead to serious complications and potentially fatal outcomes (10).

Conclusion

Accurate global, regional, and national estimates of diabetes prevalence are crucial for the planning and monitoring of prevention and treatment strategies, as well as for evaluating progress toward the goals set by joint global action plans for non-communicable disease control and sustainable development. Based on the studies, the present study is the most comprehensive to date, examining the prevalence of diabetes in Iranian pilgrims over the past decade, encompassing about half a million Iranian pilgrims. The results of the study reveal a significant variation in the prevalence of

diabetes across different age groups, genders, and provinces. However, there appears to be a dearth of in-depth information on lifestyle adaptations and behavioral determinants among Hajj pilgrims concerning the management of chronic diseases during the Hajj. Therefore, appropriate screening, diagnosis, and management by primary care physicians are essential to prevent adverse health outcomes and mitigate the economic burden.

Declaration of Competing Interest:

The authors declare that they have no known competing financial interests or personal relationships that could have appeared to influence the work reported in this paper.

Funding: This research received no external funding.

Consent Statement: Not applicable.

Acknowledgements:

We appreciate the Hajj Pilgrimage Medical Centre, Iranian Red Crescent Society for offering the data used in our study. We also thank the respected reviewers who improved the quality of the article with their valuable comments. The data analyzed in this study can be accessed by sending a request to the corresponding author and the Hajj Pilgrimage Medical Centre, Iranian Red Crescent Society.

Data Availability Statement:

CRediT authorship contribution statement

Pirhossein Kolivand: Conceptualization, Project administration.

Peyman Saberian: Conceptualization, Writing – review & editing.

Samad Azari: Conceptualization, Data curation, Writing – review & editing.

Fereshteh Karimi: Data curation, Writing – original draft.

Soheila Rajaei: Data curation, Data analysis.

Hossein Saffari: Data curation, Writing – review & editing.

Masoud Behzadifar: Methodology, Visualization, Writing – review & editing.

Arash Parvari: Methodology, Visualization, formal analysis.

Ali Marashi: Conceptualization, Writing – review & editing, Investigation.

Taher Doroudi: Data curation, Conceptualization.

Seyed Jafar Ehsanzadeh: Writing – review & editing.

Behzad Raei: Writing – review & editing.

References

.1 Freitag U. The History of the Hajj as Heritage: Asset or Burden to the Saudi State? Travelling Pasts: The Politics of Cultural Heritage in the Indian Ocean World: Brill; 2019. p. 62-79.

.2 Shirah BH, Al Nozha FA, Zafar SH, Kalumian HM. Mass gathering medicine (Hajj pilgrimage in Saudi Arabia): The outcome of cardiopulmonary resuscitation during Hajj. Journal of Epidemiology and Global Health. 2019;9(1):71.

.3 Al-Asmary S, Al-Shehri A-S, Abou-Zeid A, Abdel-Fattah M, Hifnawy T, El-Said T. Acute respiratory tract infections among Hajj medical mission personnel, Saudi Arabia. International Journal of Infectious Diseases. 2007;11(3):268-72.

.4 Ong KL, Stafford LK, McLaughlin SA, Boyko EJ, Vollset SE, Smith AE, et al. Global, regional, and national burden of diabetes from 1990 to 2021, with projections of prevalence to 2050: a systematic analysis for the Global Burden of Disease Study 2021. The Lancet. 2023.

.5 Chan JC, Lim L-L, Wareham NJ, Shaw JE, Orchard TJ, Zhang P, et al. The Lancet Commission on diabetes: using data to transform diabetes care and patient lives. The Lancet. 2020;396(10267):2019-82.

.6 Yuen L, Saeedi P, Riaz M, Karuranga S, Divakar H, Levitt N, et al. Projections of the prevalence of hyperglycaemia in pregnancy in 2019 and beyond: Results from the International Diabetes Federation Diabetes Atlas. Diabetes research and clinical practice. 2019;157:107841.

.7 Saeedi P, Petersohn I, Salpea P, Malanda B, Karuranga S, Unwin N, et al. Global and regional diabetes prevalence estimates for 2019 and projections for 2030 and 2045: Results from the International Diabetes Federation Diabetes Atlas. Diabetes research and clinical practice. 2019;157:107843.

.8 Atlas D. International diabetes federation. IDF Diabetes Atlas, 7th edn Brussels, Belgium: International Diabetes Federation. 2015;33.(2)

.9 Shaikh S, Ashraf H, Shaikh K, Iraqi H, Ndour Mbaye M, Kake A, et al. Diabetes Care During Hajj. Diabetes Therapy. 2020;11:2829-44.

.10 Ibrahim M, Abdelaziz SI, Almagd MA, Alarouj M, Annabi FA, Armstrong DG ,et al. Recommendations for management of diabetes and its complications during Hajj (Muslim pilgrimage). BMJ Open Diabetes Research & Care. 2018;6.(1)

.11 Khan ID, Khan SA, Asima B, Hussaini SB, Zakiuddin M, Faisal F. Morbidity and mortality amongst Indian Hajj pilgrims: A 3-year experience of Indian Hajj medical mission in mass-gathering medicine. Journal of infection and public health. 2018;11(2):165-70.

.12 Memish ZA, Stephens GM, Steffen R, Ahmed QA. Emergence of medicine for mass gatherings: lessons from the Hajj. The Lancet infectious diseases. 2012;12(1):56-65.

.13 Khamseh ME, Sepanlou SG, Hashemi-Madani N, Joukar F, Mehrparvar AH, Faramarzi E, et al. Nationwide prevalence of diabetes and prediabetes and associated risk factors among Iranian adults: analysis of data from PERSIAN cohort study. Diabetes Therapy. 2021;12:2921-38.

.14 Khodakarami R, Abdi Z, Ahmadnezhad E, Sheidaei A, Asadi-Lari M. Prevalence, awareness, treatment and control of diabetes among Iranian population: results of four national cross-sectional STEPwise approach to surveillance surveys. BMC Public Health. 2022;22(1):1216.

.15 Najafipour H, Farjami M, Sanjari M, Amirzadeh R, Shadkam Farokhi M, Mirzazadeh A. Prevalence and incidence rate of diabetes, pre-diabetes, uncontrolled diabetes, and their predictors in the adult population in southeastern Iran: findings from KERCADR study. Frontiers in public health. 2021;9:611652.
.16 Azadnajafabad S, Ahmadi N, Rezaei N, Rashidi M-M, Saeedi Moghaddam S, Mohammadi E, et al. Evaluation of the diabetes care cascade and compliance with WHO global coverage targets in Iran based on STEPS survey 2021. Scientific Reports. 2023;13(1):13528.
.17 Yezli S, Mushi A, Almuzaini Y, Balkhi B, Yassin Y, Khan A. Prevalence of diabetes and hypertension among Hajj pilgrims: a systematic review. International journal of environmental research and public health. 2021;18(3):1155.
.18 Mousavi J, Jafari F. Prevalence of diseases in pilgrims referring to Iranian clinics in Iraq. International Journal of Travel Medicine and Global Health. 2016;4(1):31-3.
.19 Razavi SM, Sabouri-Kashani A, Ziaee-Ardakani H, Tabatabaei A, Karbakhsh M, Sadeghipour H, et al. Trend of diseases among Iranian pilgrims during five consecutive years based on a Syndromic Surveillance System in Hajj. Medical journal of the Islamic Republic of Iran. 2013;27(4):179.
.20 Gautret P, Bauge M, Simon F, Benkouiten S, Valéro R, Parola P, et al. Overweight and obesity in French Hajj pilgrims. Journal of immigrant and minority health. 2013;15:215-8.
.21 Roglic G. WHO Global report on diabetes: A summary. International Journal of Noncommunicable Diseases. 2016;1(1):3-8.
.22 Gaddoury MA, Armenian HK. Epidemiology of hajj pilgrimage mortality: Analysis for potential intervention. Journal of Infection and Public Health. 2023.
.23 Ogurtsova K, da Rocha Fernandes J, Huang Y, Linnenkamp U, Guariguata L, Cho NH, et al. IDF Diabetes Atlas: Global estimates for the prevalence of diabetes for 2015 and 2040. Diabetes research and clinical practice. 2017;128:40-50.
.24 Hashim S, Ayub ZN, Mohamed Z, Hasan H, Harun A, Ismail N, et al. The prevalence and preventive measures of the respiratory illness among Malaysian pilgrims in 2013 Hajj season. Journal of travel medicine. 2016;23(2):tav019.
.25 Al-Masud SMR, Bakar AA ,Yussof S. Determining the types of diseases and emergency issues in Pilgrims during Hajj: A literature review. Stat Inf. 2016;5.(7)
.26 Mushi A, Yassin Y, Khan A, Alotaibi B, Parker S, Mahomed O, et al. A longitudinal study regarding the health profile of the 2017 South African Hajj pilgrims. International Journal of Environmental Research and Public Health. 2021;18(7):3607.
.27 Ardiana M, Paramitha AD, Shabrina FA, Harsoyo PM. Risk Factors of Hospitalization and Mortality among Hajj Pilgrims in Indonesia. 2023.
.28 Ebrahimi H, Emamian MH, Hashemi H, Fotouhi A. High incidence of diabetes mellitus among a middle-aged population in Iran: a longitudinal study. Canadian journal of diabetes. 2016;40(6):570-5.
.29 Johari MG, Jokari K, Mirahmadizadeh A, Seif M ,Rezaianzadeh A. The prevalence and predictors of pre-diabetes and diabetes among adults 40–70 years in Kharameh cohort study: A population-based study in Fars province, south of Iran. Journal of Diabetes & Metabolic Disorders. 2022:1-11.
.30 Hasan G, Moabber H, Alyamani A, Sayeed A, Altatar F. Study on risk factors (predisposing factors) for poor diabetes control during Hajj (1436/2015) in people with diabetes. Pakistan journal of medical sciences. 2016;32(5):1092.

Figure 1

Click here to access/download;Figure;Figure 1.docx

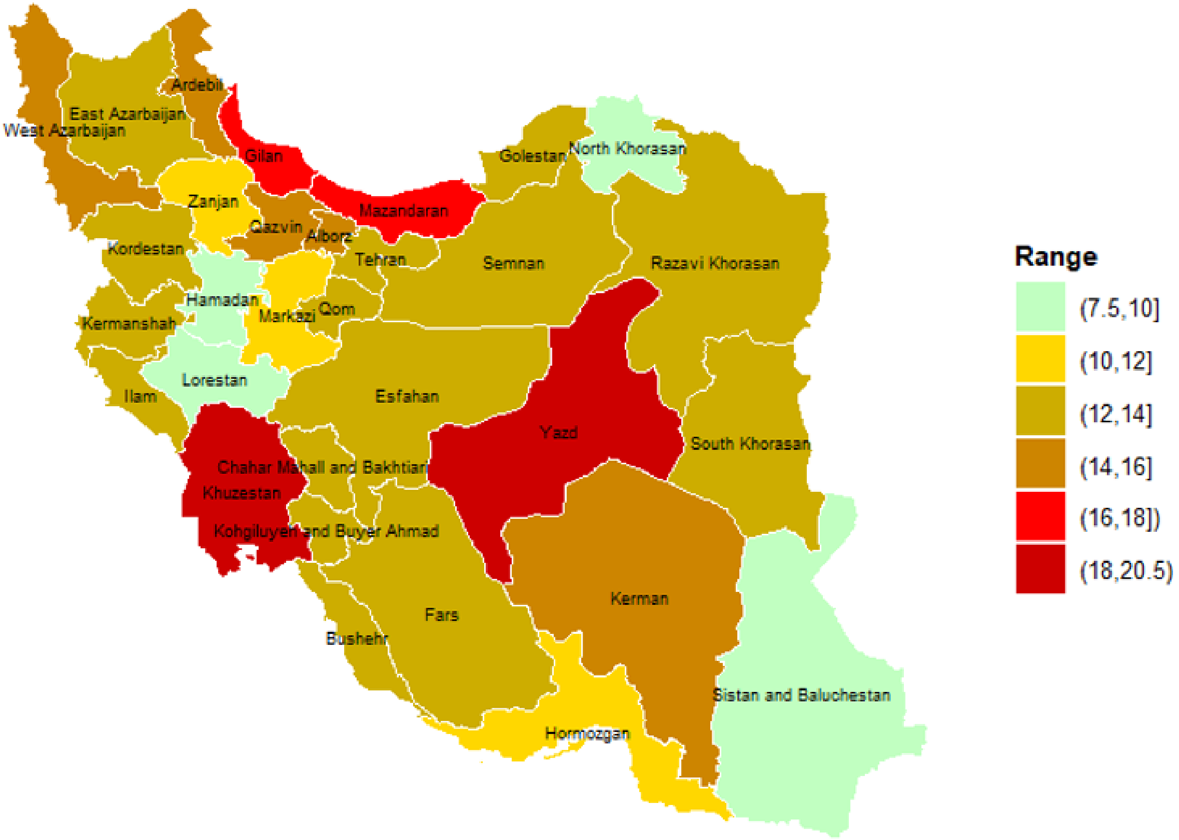

Figure 1 Prevalence (%) of DM by province

Figure 2 Click here to access/download;Figure;Figure 2.docx

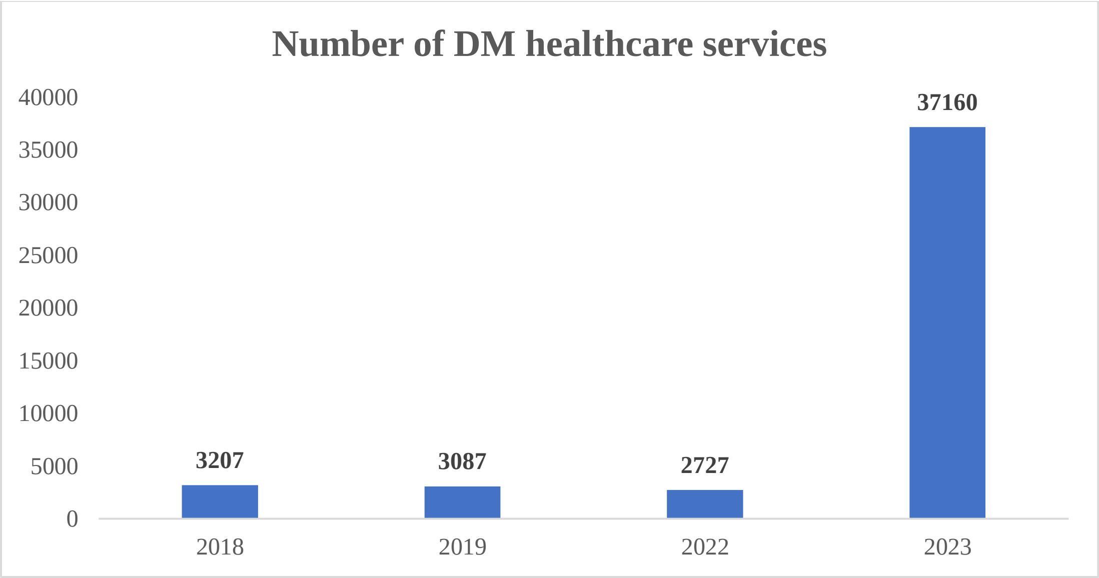

Figure 2: Trend of DM Healthcare delivery

original ethics approval letter

Click here to access/download
Supporting Information
original ethics approval letter.pdf
