# Supplementary material for: Patterns of diabetes mellitus by age, sex, and province among Iranian Hajj pilgrims and health care delivery during 2012–2022: A retrospective study of 469,581 participants
Source: PLoS One. 2024 Oct 8;19(10):e0311399. doi: 10.1371/journal.pone.0311399 (PMC11460693; doi:10.1371/journal.pone.0311399)
Supplement: Attachment — Submitted filename: PONE-D-24-05546.docx [file pone.0311399.s002.docx]

Manuscript require major modifications to eradicate the ambiguity among the sections.

**Introduction:**

The selection of Hajj pilgrims as the study population should be detailed in the introduction to highlight its significance.

Signify the importance of the prevalence of diabetes among haj pilgrims, as it is a non-communicable disease.

As mentioned, “diabetes is prevalent in Iran,” and the age of pilgrims mentioned is 40 and more. So, by the age of 40, diabetes is usual worldwide, thus nullifying the significance of the study.

As written “Limited studies have evaluated the epidemiological pattern of diabetes and the medical care required and provided to pilgrims.” Unclear statement & provide reference of those limited studies.

The unclear rationale of the study.

**Methodology**:

Methodology should be organized as study setting, study design, study type and so on, to clear the section. More depth and details are required.

As written, “Due to the retrospective nature of the study claims, data were analyzed anonymously, and written informed consent was waived…………………………” When the study is retrospective, the data is obtained from a secondary source (of 10 years), and patients are not involved, how is consent obtained?

What confounding factors were considered during data extraction?

If no sampling is done in the work, remove “sampling” from the heading. Describe the data fetching method from records by clearly describing the inclusion and exclusion criteria. “We also excluded Pilgrims whose information was not fully recorded in the databases” is an insufficient exclusion criterion.

“Data were accessed for research purposes on 25.04.2023”. As the date is given in particular, arise the question that entire information of thousands of sample is obtained in one single day??

Age category -four groups are mentioned starting from 15 years, while in abstract the age of hajj pilgrims are mentioned as “about 45-70 yrs”. Kindly rectify it .

The whole manuscript requires grammar corrections. Words like “we”, “us” and “our” are suggested not to be used.

Data analysis section is not suitable to mention the clusters. As clusters is an essential part, it should be described in detail in the study settings. However statistical tests should only be mentioned in “data analysis section”.

**Results:**

The text as the description of tables does not align with the values. The use of “ about” is discouraged as the discrete values are mentioned.

The description of the tables should be done in an orderly manner as the appearance of every item: Like mention age first than year. Rewrite the text of table 1.

Table 2. details are required about N(%) in the footnote. Age groups should be written similarly as in table 1.

“Prevalence of DM by province” does not describe provinces in the methodology section.

“As indicated by the data in the graph,” it is not a graph; it is a map. Also, it requires a number, no matter if it is just one.

Table 3: 0 and 1 should be described in the footnote. Also, in the methodology section.

Table 4: Improper presentation of data.

DM Healthcare Delivery and Economic Burden: the country should be mentioned as Iran or Saudi Arabia?

**Discussion:** Comparisons are done with the results of entirely different study settings: Check ref 13 and 14.

“These findings underscore the significant impact of age on the prevalence of DM among Iranian Hajj pilgrims.”- The finding is historic, the study subjects were 40 years and above, making the prevalence of diabetes mellitus obvious.

Very old references are used just for the purpose of comparison and not for the standardized procedure. (2007, many from 2016,2017).

**Conclusion from the Reviewer:** the study lacks depth in methodology, and lacks many details. Should be meticulously revised before submitting. In this stage the manuscript is not suitable for acceptance and will affect the reputation of journal.
