# Supplementary material for: Patterns of diabetes mellitus by age, sex, and province among Iranian Hajj pilgrims and health care delivery during 2012–2022: A retrospective study of 469,581 participants
Source: PLoS One. 2024 Oct 8;19(10):e0311399. doi: 10.1371/journal.pone.0311399 (PMC11460693; doi:10.1371/journal.pone.0311399)
Supplement: Attachment — Submitted filename: Response to the Reviewers.docx [file pone.0311399.s004.docx]

***Dear Editor,***

The authors of the article have carefully checked the comments of the Reviewers and made the necessary changes in the article. These changes have been highlighted in the text. Additionally, the responses have been presented in the below table. We would like to express our gratitude to the referees for their valuable contributions, which have significantly enhanced the quality of the article.

Respectfully,

[Dr Samad Azari]

Table 1- The Journal Requirements

| **Num** | **Comment** | **Response** |
| --- | --- | --- |
| 1 | Please ensure that your manuscript meets PLOS ONE's style requirements | Thanks for your reminder, all the text was reviewed according to the journal guidelines and the necessary corrections were made. |
| 2 | Please provide a complete Data Availability Statement in the submission form | Data Availability Statement was added. |
| 3 | We note that Figure 1 in your submission contain map images which may be copyrighted. All PLOS content is published under the Creative Commons Attribution License (CC BY 4.0), which means that the manuscript, images, and Supporting Information files will be freely available online, and any third party is permitted to access, download, copy, distribute, and use these materials in any way, even commercially, with proper attribution. For these reasons, we cannot publish previously copyrighted maps or satellite images created using proprietary data, such as Google software (Google Maps, Street View, and Earth). For more information, see our copyright guidelines: http://journals.plos.org/plosone/s/licenses-and-copyright | It should be mentioned that in order to draw the figure in this manuscript, we used R software version 4.3.1 and the following packages under their respective licenses: `raster` (GPL-3), `geodata` (GPL-3), ` rgeos` (GPL-2), `ggplot2` (MIT), `dplyr` (MIT), and `readxl` (MIT). We acknowledge and thank the developers of these packages for their contributions to the open-source community.  We must emphasize that the presented map was not copied from any other source and there was no need to obtain permission from any original source. Therefore, we adhere to the Creative Commons Attribution 4.0 International (CC BY) license and presenting the map of this research has no conflict with the CCBY license and according to journal rules, the manuscript, images, and Supporting Information files will be freely available online, and any third party is permitted to access, download, copy, distribute, and use these materials in any way, even commercially, with proper attribution. |

| Table 2- The Responses to The Reviewer’s Comments | | |
| --- | --- | --- |
| **Num** | **Comment** | **Response** |
| 1 | Reviewer #1:   1. Major revisions are required. 2. contradictions in results. 3. the study lacks depth in methodology, and lacks many details. 4. Should be meticulously revised before submitting. In this stage the manuscript is not suitable for acceptance and will affect the reputation of journal. | Thanks to the precious comments of the respected reviewer.  This descriptive and analytical study examines the information related to all Iranian Hajj pilgrims over ten years with more than half a million people's data. This survey includes many Variables Such as Age, Gender, Province, Diabetes Status, Blood Pressure, Insulin Use, and healthcare delivery. Therefore, in addition to the long study period that increases the reliability of the results, this study has also examined various important variables. These results can be used by the country's health system policymakers and also can be used by interested organizations such as the Red Crescent Society or the Ministry of Health in changing and improving screening and intervention policies related to Hajj. However, according to the valuable opinions of the reviewers, changes were made to the study method at this stage. The data analysis section was rewritten. Additionally, more details were provided in the study results section. In this part, the results related to the use of different types of insulin and their doses were also presented. Accordingly, the discussion section was also modified.  Thank you again for your valuable comments. The research team welcomes your expert feedback. Undoubtedly, your comments will improve the quality of this research. |
| 2 | Reviewer #2: There is good data in this study. Some comments:  1. Indicate why 2020/2021 data is not available (we know its due to COVID-19)  2. Is there any indication of %age pilgrims who used insulin?  3. If insulin usage is known was the pathology/morbidity increased in that group?  4. Any indication of knowledge of insulin storage/handling during travel? (There was a recent study by Yezli on it). | Thanks for the valuable comments of the respected reviewer.  As the respected reviewer has correctly pointed out, the data of some years were excluded in this study. This information was related to the Mina accident in 2015 and the information related to period of the Covid-19 pandemic. According to the honourable comment of the reviewer, relevant explanations were added in the methodology section.  Regarding the reviewer's comment about insulin use, the desired section was added to the study findings section. In this section, the research findings on the units and types of insulin used among pilgrims are presented. Also, the valuable topics about insulin mentioned by the referee were examined in the discussion section of the study.  Thank you again for your valuable comments. These comments led to the improvement of research quality. |
