# Supplementary material for: Patterns of diabetes mellitus by age, sex, and province among Iranian Hajj pilgrims and health care delivery during 2012–2022: A retrospective study of 469,581 participants
Source: PLoS One. 2024 Oct 8;19(10):e0311399. doi: 10.1371/journal.pone.0311399 (PMC11460693; doi:10.1371/journal.pone.0311399)
Supplement: Attachment — Submitted filename: Review.docx [file pone.0311399.s006.docx]

Overall, the is article is so unique and has an important outcome that worth to be published. Minor modification could enrich the results and strengthens the findings of the study.

- Introduction:
  - Hajj is attracting about 2.5 million Hajj pilgrims. NOT 3.5.
- Methodology:
  - More information about the database is required:
    - Were the data was available for all Iranian pilgrims?
    - What are the medical data?
    - There was a mention about FBS in the results. More information is required about such data and how and when was collected?
    - What other information in database related to health records available can be added to this analysis?"
- Results:
  - Were there any data about the compliance of diabetic pilgrims to treatment?
  - When mentioning the rate of receiving DM services, provide the following:
    - percentage out of the total provided services NOT numbers each year.
    - percentage of dietetic pilgrims required services out of all dietetic pilgrims.
    - Detailed information about the medical services attained by diabetic patients would be very helpful? Incidence of specific complications, Acquiring extra medications, modification of treatment?"
- Table 5:
  - Detailed information need to be added about the acquisition of FBS. Was it collected from all pilgrims? Was it collected once? and when that was? Is there any follow-up data?"
- Discussion:
  - It is better to emphasize the main finding of the study briefly in the first paragraph, then discuss the findings in following paragraphs.
