# Supplementary material for: Patterns of diabetes mellitus by age, sex, and province among Iranian Hajj pilgrims and health care delivery during 2012–2022: A retrospective study of 469,581 participants
Source: PLoS One. 2024 Oct 8;19(10):e0311399. doi: 10.1371/journal.pone.0311399 (PMC11460693; doi:10.1371/journal.pone.0311399)
Supplement: Attachment — Submitted filename: Responces to Review-1.docx [file pone.0311399.s007.docx]

Thanks to the valuable comments of the respected reviewers, the answers to the comments are provided in this file. In the introduction section, corrections were made. In the method section, the necessary parts were added. In the findings section, the comments were answered. In the discussion section, the items desired by the reviewers were applied. Also, the details of some studies were added and reference 16 was removed.

**Reviewer #2**: The minor comments in the initial draft were NOT addressed. I have put it in Word format in the attached document.
Some of these include:
1. The word 'ceremony' should be deleted.

**Answer**: Thank you for your valuable comments. revised.

2. India is NOT a Muslim majority country.

**Answer**: T Thank you for your valuable comments. his item was checked. India is also one of the ten countries that has the most Hajj pilgrims. Related articles are provided below.

- The prevalence of diabetes is high in several nations with large Muslim population, such as Pakistan, Indonesia, Egypt, Bangladesh and India, and all of these countries rank amongst the top ten countries in the world in terms of diabetes prevalence
- Shaikh S, Ashraf H, Shaikh K, Iraqi H, Ndour Mbaye M, Kake A, Mohamed GA, Selim S, Wali Naseri M, Syed I, Said JA. Diabetes care during Hajj. Diabetes Therapy. 2020 Dec; 11:2829-44.

3. Hajj WAS held in 2015, 2020 and 2021. Please correct that.

**Answer**: Thank you for your valuable comments. This item was corrected. It should be noted that there was a mistake in converting the date.

4. Iranian pilgrims DID attend 2015 Hajj. I met a number of them.

**Answer**: Thank you for your valuable comments. This item was corrected. It should be noted that there was a mistake in converting the date.

5. Iranian pilgrims did not attend the 2016 Hajj.

**Answer**: Thank you for your valuable comments. This item was corrected. It should be noted that there was a mistake in converting the date.

6. The paragraph below is not clear. Your study was till 2022, yet you include 2023. Does the below paragraph refer only to Iranians and only dialysis and why the massive difference for 2023?

**Answer**: No, we mean all health services provided, including general and specialized visits, medicine and prescription, nursing, relief and transportation, etc., which are available and reported in 2018, 2019, 2022. The part related to 2023 was deleted.

Please clarify. Did 2727 Iranian pilgrims need dialysis in 2022 and 37160 need it 2023? Do these numbers refer to Iranian pilgrims only? Please rewrite.

**Answer**: It means all services that were provided to diabetic pilgrims. This part has been rewritten.

**Reviewer #3:** 

Introduction:

- Hajj is attracting about 2.5 million Hajj pilgrims. NOT 3.5.

**Answer**: This item was corrected in the article.

Check currency. None of the suggestions in my first review have been incorporated. Was the PDF that I sent checked?

**Answer**: This item was corrected in the article.

India is not a muslim amjority country

**Answer**: This item was checked. India is also one of the ten countries that has the most Hajj pilgrims. Related articles are provided below.

- The prevalence of diabetes is high in several nations with large Muslim population, such as Pakistan, Indonesia, Egypt, Bangladesh and India, and all of these countries rank amongst the top ten countries in the world in terms of diabetes prevalence
- Shaikh S, Ashraf H, Shaikh K, Iraqi H, Ndour Mbaye M, Kake A, Mohamed GA, Selim S, Wali Naseri M, Syed I, Said JA. Diabetes care during Hajj. Diabetes Therapy. 2020 Dec; 11:2829-44.

Check currency. None of the suggestions in my first review have been incorporated. Was the PDF that I sent checked?

**Answer**: This item was corrected in the article.

- Methodology:
  - More information about the database is required:
    - Were the data was available for all Iranian pilgrims?
    - What are the medical data?
    - There was a mention about FBS in the results. More information is required about such data and how and when was collected?
    - What other information in database related to health records available can be added to this analysis?"

**Answer**: This description was added to the method section» The Islamic Republic of Iran's Red Crescent is responsible for conducting thorough medical examinations of Iranian Hajj pilgrims prior to their departure to Saudi Arabia. This screening follows a specific protocol established by the Medical Board of the Red Crescent, which includes clinical and para-clinical tests to create health profiles for each pilgrim. These profiles assess for various health conditions such as cardiac diseases, hypertension, respiratory diseases, diabetes, and psychiatric disorders. Additionally, influenza and pneumococcal vaccinations are administered to all pilgrims 15 days before their trip. The Red Crescent also implements a syndromic surveillance system to monitor the health of pilgrims throughout their journey. This system tracks the health status of pilgrims, focusing on 19 specific diseases. When pilgrims experience health issues during the pilgrimage, they are first examined by physicians in their travel caravans, with referrals to Iranian hospitals in Mecca and Medina if necessary. Physicians in the caravans can perform essential lab tests as needed. The health data collected during these processes is crucial for identifying disease trends and planning future health measures for Hajj pilgrims«.

- Results:
  - Were there any data about the compliance of diabetic pilgrims to treatment?
  - When mentioning the rate of receiving DM services, provide the following:
    - percentage out of the total provided services NOT numbers each year.
    - percentage of dietetic pilgrims required services out of all dietetic pilgrims.
    - Detailed information about the medical services attained by diabetic patients would be very helpful? Incidence of specific complications, Acquiring extra medications, modification of treatment?"

**Answer**: Detailed information about the medical services obtained by diabetic patients, such as the special complications, receiving additional drugs, treatment modification, should be done by checking the patients' files case by case. Therefore, access to the desired information of the respected reviewer was not possible in this study. We hope that future studies will investigate the desired aspects. Also, because the information about services was not available for all years, we could not provide the percentage of services provided in each year in this section.

- Table 5:
  - Detailed information needs to be added about the acquisition of FBS. Was it collected from all pilgrims? Was it collected once? and when that was? Is there any follow-up data?"

**Answer**: Screening of pilgrims before travel includes clinical and paraclinical tests to create a health profile for each pilgrim. In this screening, various health conditions are evaluated. When pilgrims experience health problems during the Hajj journey, they are first examined by physicians in their traveling caravans and, if necessary, refer to Iranian hospitals in Mecca and Medina. Doctors in the caravans can perform necessary laboratory tests if needed. Information about treatment complexity, treatment compliance, and other information for each individual is recorded in their file. It is possible to access this information of patients in the form of file reading and case by case. Therefore, access to the desired information of the respected reviewer was not possible in this study. We hope that future studies will investigate the desired aspects.

Fig 3:

Should range not be written as 7.5-10 then 10-12?

- **Answer**: This item was corrected in the article.
- Discussion:
  - It is better to emphasize the main finding of the study briefly in the first paragraph, then discuss the findings in following paragraphs.
- **Answer**: This item was corrected in the article. Also, the items desired by the reviewers were applied. Also, the details of some studies were added and reference 16 was removed.
