# Supplementary material for: Patterns of diabetes mellitus by age, sex, and province among Iranian Hajj pilgrims and health care delivery during 2012–2022: A retrospective study of 469,581 participants
Source: PLoS One. 2024 Oct 8;19(10):e0311399. doi: 10.1371/journal.pone.0311399 (PMC11460693; doi:10.1371/journal.pone.0311399)
Supplement: Attachment — Submitted filename: DM Manuscript clean revise Reviewer comments.docx [file pone.0311399.s005.docx]

**Patterns of Diabetes Mellitus by Age, Sex, and Province among Iranian Hajj Pilgrims and Health Care Delivery during 2012-2022: A Retrospective Study of 469,581 Participants**

Pirhossein Kolivand^1†^, Peyman Saberian^2†^, Hossein Saffari^3^, Taher Doroudi^4,5^, Ali Marashi^4^, Masoud Behzadifar^6^, Fereshteh Karimi^7^, Soheila Rajaei^7^, Behzad Raei^8^, Seyed Jafar Ehsanzadeh^9^, Arash Parvari^10^, Samad Azari^11,7^

1: Department of Health Economics, Faculty of Medicine, Shahed University, Tehran, Iran.

2: Department of Anesthesiology, Imam Khomeini Hospital Complex, Tehran University of Medical Sciences, Tehran, Iran.

3: Hasheminejad Kidney Center (HKC), Iran University of Medical Sciences (IUMS), Tehran, Iran.

4: Iranian Red Crescent Society, Haj Medical Center. Tehran, Iran.

5: Shefa Neuroscience Research Center, Khatam Al anbia Hospital. Tehran, Iran.

6: Social Determinants of Health Research Center, Lorestan University of Medical Sciences, Khorramabad, Iran.

7: Research Center for Emergency and Disaster Resilience, Red Crescent Society of the Islamic Republic of Iran, Tehran, Iran.

8: Razi Educational and Therapeutic Center, Tabriz University of Medical Science, Tabriz, Iran.

9: English Language Department, School of Health Management and Information Sciences, Iran University of Medical Sciences, Tehran, Iran.

10: Department of Epidemiology and Biostatistics, School of Public Health, Tehran University of Medical Sciences, Tehran, Iran.

11: Hospital Management Research Center, Health Management Research Institute, Iran University of Medical Sciences, Tehran, Iran.

† Contributed equally as the first authors

Corresponding author: Samad Azari Email: [Samadazari1010@gmail.com](mailto:Samadazari1010@gmail.com)

**Abstract**
**Objective:** Hajj is among the oldest pilgrimages in the world, there is a limited study that evaluates the epidemiological pattern of Diabetes Mellitus (DM) and the medical care required and provided to pilgrims. The present study assessed the prevalence and pattern of DM in Iranian pilgrims from 2012-22.

**Method:** All demographic information, risk factors, and the prevalence of DM were extracted from the database and medical records of the Hajj Pilgrimage Medical Centre, Iranian Red Crescent Society through file reading. Also, to investigate the effect of the risk factors considered in the study, the multiple logistic regression model was used.

**Results:** The present study included data from 469,581 Hajj pilgrims. Most pilgrims were in the age group of 45 to 70 years (73.25%). The prevalence of diabetes in patients over 70 years old was the highest (16.73%). The prevalence of DM was estimated at 14.64 % in women and 12.51 % in men. The lowest DM prevalence was in Lorestan (7.81%), North Khorasan (9.07%), Sistan and Baluchistan (9.29%), and Hamedan (9.41), respectively. The highest prevalence rate was in Khuzestan (20.12%), Yazd (19.14), and Mazandaran (17.55), respectively. Our analysis reveals that, for instance, with each yearly increase in the age of the pilgrims (assuming other variables remain constant), the odds of having DM increase by 0.04. For the gender, the odds of having DM among women is 0.33 higher than among men, when the other variable is constant.

**Conclusions**: The study results show a significant difference in the prevalence of diabetes in age, gender, and distribution in different provinces. Therefore, appropriate screening, diagnosis, and management by primary care physicians are necessary to prevent adverse health outcomes and reduce the economic burden of mortality and morbidity.

**Keywords:** Diabetes Mellitus, Hajj, Prevalence**,** Iran

1. **Introduction**

The Hajj, an annual pilgrimage, is one of the largest mass gatherings worldwide, attracting over 3.5 million pilgrims from 200 countries to perform specific rituals in Mecca, the Kingdom of Saudi Arabia (KSA). Successful participation in this ritual requires meticulous preparations undertaken months in advance. The increasing number of pilgrims challenges global and public health security for KSA and countries of various nationalities and races. These challenges encompass housing, food, water, transportation, communication, sanitation, mass-gathering and crowd control, and security, with the density of people reaching up to 7 individuals per square meter(1, 2).

Diabetes Mellitus (DM) is a serious, chronic disease characterized by elevated blood glucose concentrations due to the effects of abnormal β-cell biology on insulin action. DM is one of the leading causes of death and disability worldwide, affecting individuals regardless of their country, age group, or sex. In 2017, the expenses incurred by health systems due to diabetes were estimated to be 727 billion dollars. The number of people suffering from diabetes was estimated to be 285 million in 2009, 415 million in 2015, and 529 million (95% uncertainty interval [UI] 500–564) in 2021, indicating a continuous increase in global cases(3-5). The prevalence of diabetes is high in several countries with a majority or large Muslim population, such as Pakistan, Indonesia, Egypt, Bangladesh, Iran, and India. All these countries rank among the top ten in the world in terms of diabetes prevalence. Based on a global data analysis, it is estimated that 148 million Muslims are affected by diabetes(6, 7).

Mass-gathering medicine at Hajj is challenged by issues of high morbidity, healthcare accessibility, patient management, and evacuation, especially in emergencies(8, 9). Limited studies have evaluated the epidemiological pattern of diabetes and the medical care required by, and provided to pilgrims. The present study evaluated the prevalence and pattern of DM in Iranian Hajj pilgrims during the years 2012-22. Given the high population of pilgrims in the study, the results can be used to develop preventive and health protocols. This study will provide a comprehensive overview of diabetes status in Iranian pilgrims over ten years.

2. **Methodology**

**2.1 Ethics approval and consent to participate**

This study is a component from the research project with the Code of Ethics IR.RCS.REC.1401.023 from the Iranian Red Crescent Society, which has been conducted at the Research Center for Emergency and Disaster Resilience, Red Crescent Society of the Islamic Republic of Iran, Tehran, Iran. Due to the retrospective nature of the study claims, data were analyzed anonymously, and written informed consent was waived according to the Research Ethics Committee of the Iranian Red Crescent Society before the start of the study. All stages of the study, including the design, implementation, and reporting, were conducted without any involvement from patients or the public. All methods were carried out according to Helsinki guidelines and regulations.

**2.2 Research Method**

The present study was conducted in 2023. In this study, the prevalence of DM was examined by gender, province, and year from 2012 to 2022. All demographic information, risk factors, and the prevalence of diabetes were extracted from the databases of the Hajj and Pilgrimage Medical Center through file reading. Overall, this study investigated the age distribution of pilgrims, the prevalence of DM by age and gender among pilgrims in each age group, and the prevalence of DM among pilgrims by province and year.

**2.3 Sampling and Inclusion & Exclusion criteria:**

No sampling was made in this study, and all Hajj pilgrims during the study period (from 2012 to 2022) were included in the analysis. We excluded Pilgrims whose information was not fully recorded in the databases. Data were accessed for research purposes on 25.04.2023. There is hence no data for those years.

**2.4 Age Category:**

In the present study, pilgrims were categorized into four age groups, under 15 years, 15-44 years, 45-69 years, and over 70-year-old pilgrims, respectively.

**2.5 Data Analysis and Statistical Tests**

The extracted data was analyzed using Stata 17. The figure was generated by R software version 4.3.1 with the raster, geodata, rgeos, ggplot2, dplyr, and readxl packages. All data were cleaned and checked for accuracy. Initial descriptive analyses were performed to summarize the data using frequency distributions. To investigate the effect of risk factors such as fasting blood sugar (FBS), age, and sex on the odds of diabetes among pilgrims, a multiple logistic regression model was applied. Since the diabetes status of individuals is a dichotomous variable (0 and 1), logistic regression was deemed an appropriate model for this study. Independent variables included in the model were selected based on clinical relevance and prior research findings. Considering that the prevalence of DM among pilgrims varies across provinces, and pilgrims within the same province are more similar to each other, the province was treated as a clustering variable. Robust standard errors were used to adjust for the correlation between pilgrims within the same province, accounting for intra-cluster correlation. All statistical analyses were conducted with a predetermined significance level of 5% (α = 0.05).

3. **Results:**

**3.1 Characteristics of the study sample**

In total, the present study included data from 469,581 hajj pilgrims, about 50% females and 50% males. Of the total pilgrims, 20.92% were hypertensive, and 13.55% had DM. The highest number of pilgrims, 89,492 individuals, belonged to the year 2019, and 73.25% were in the group aged 45–70 years. The age average was 55.48 ± 11.24 years (Table 1).

**Table 1.** Characteristics of Iranian Hajj pilgrims

| (%)N | **Variables** |
| --- | --- |
|  | **Gender** |
| 234,773 (49.99%) | Male |
| 234,808 (50.01%) | Female |
| **Age** (years) | |
| 133 (0.02%) | <15 |
| 75,063 (15.98%) | 15-44 |
| 343,977 (73.25%) | 45-69 |
| 50,408 (10.75%) | 70 $\geq$ |
| 55.48 ± 11.24 | Mean ± SD |
| **Diabetes** | |
| 63,637 (13.55%) | Yes |
| 405,944 (86.45%) | No |
| **Hypertension** | |
| 98,264 (20.92%) | Yes |
| 371,317 (79.08%) | No |
| **Year** | |
| 61,456 (13.09%) | 2012 |
| 61,364 (13.07%) | 2013 |
| 61,853 (13.17%) | 2014 |
| 71,053 (15.13%) | 2016 |
| 85,416 (18.19%) | 2017 |
| 89,492 (19.06%) | 2019 |
| 38,947 (8.29%) | 2022 |

**3.2 Distribution of DM by Gender and Age**

According to the findings, the prevalence of DM among pilgrims increases with age, with the highest prevalence of the disease observed in pilgrims aged 70 years and above (Table 2). In terms of gender, 46% of diabetic patients were men and 54% were women.

**Table 2.** Distribution of DM in hajj pilgrims by Age and Gender

| **Total** | **DM, N (%)** | | **Age (years)** |
| --- | --- | --- | --- |
| - | Yes | No |  |
| 133 | 0(0.0%) | 133 | <15 |
| 74,972 | 2,211(2.94%) | 72,761 | 15≤age<45 |
| 343,503 | 53,006(15.43%) | 290,497 | 45≤age<70 |
| 50,339 | 8,420(16.72%) | 41,919 | 70 $\geq$ |

**3.3 Distribution of DM by province**

As indicated by the data in the graph, the prevalence of diabetes among Hajj pilgrims from Lorestan province was the lowest among the 31 provinces. Of the 7110 pilgrims who journeyed to Mecca between 2012 and 2022, only 555 were diagnosed with diabetes, resulting in an estimated disease prevalence of 7.81%. Conversely, Khuzestan province exhibited the highest prevalence of this disease. Out of 24290 pilgrims, 4888 were affected by diabetes, yielding a prevalence rate of 20.12% in this province (Table 3). These findings highlight the significant regional disparities in diabetes prevalence among Hajj pilgrims (Fig.1).

**Figure 1:** Prevalence (%) of DM by province

**Table 3.** Distribution of DM by province among Hajj pilgrims

| **Total** | **DM** | | **Province** |
| --- | --- | --- | --- |
|  | **1** | **0** |  |
| 2,493 | 314 | 2,179 | **Chahar Mahaal and Bakhtiari** |
| (100) | (12.60) | (87.40) |  |
| 857 | 119 | 738 | **Kohgiluyeh and Boyer-Ahmad** |
| (100) | (13.89) | (86.11) |  |
| 7,989 | 1,135 | 6,854 | **Alborz** |
| (100) | (14.21) | (85.79) |  |
| 4,057 | 584 | 3,473 | **Ardabil** |
| (100) | (14.39) | (85.61) |  |
| 5,394 | 733 | 4,661 | **Bushehr** |
| (100) | (13.59) | (86.41) |  |
| 15,246 | 1,982 | 13,264 | **East Azarbayjan** |
| (100) | (13.00) | (87.00) |  |
| 22,152 | 2,875 | 19,277 | **Fars** |
| (100) | (12.98) | (87.02) |  |
| 3,748 | 609 | 3,139 | **Gilan** |
| (100) | (16.25) | (83.75) |  |
| 21,724 | 2,787 | 18,937 | **Golestan** |
| (100) | (12.83) | (87.17) |  |
| 13,034 | 1,227 | 11,807 | **Hamadan** |
| (100) | (9. 41) | (90.59) |  |
| 5,594 | 656 | 4,938 | **Hormozgan** |
| (100) | (11.73) | (88.27) |  |
| 711 | 94 | 617 | **Ilam** |
| (100) | (13.22) | (86.78) |  |
| 44,020 | 6000 | 38,020 | **Esfahan** |
| (100) | (13.63) | (86.37) |  |
| 17,534 | 2,604 | 14,930 | **Kerman** |
| (100) | (14.85) | (85.15) |  |
| 5,450 | 671 | 4,779 | **Kermanshah** |
| (100) | (12.31) | (87.69) |  |
| 24,290 | 4,888 | 19,402 | **Khuzestan** |
| (100) | (20.12) | (79.88) |  |
| 8,999 | 1,093 | 7,906 | **Kurdistan** |
| (100) | (12.15) | (87.75) |  |
| 7,110 | 555 | 6,555 | **Lorestan** |
| (100) | (7.81) | (92.19) |  |
| 9,239 | 1,080 | 8,159 | **Markazi** |
| (100) | (0.14) | (99.86) |  |
| 21,237 | 3,727 | 17,510 | **Mazandaran** |
| (100) | (17.55) | (82.45) |  |
| 7,186 | 652 | 6,534 | **North Khorasan** |
| (100) | (9.07) | (90.93) |  |
| 6,408 | 960 | 5,448 | **Qazvin** |
| (100) | (14.98) | (85.02) |  |
| 13,359 | 1,649 | 11,710 | **Qom** |
| (100) | (12.34) | (87.66) |  |
| 57,586 | 8,020 | 49,566 | **Razavi Khorasan** |
| (100) | (13.93) | (87.07) |  |
| 6,573 | 866 | 5,707 | **Semnan** |
| (100) | (13.18) | (86.82) |  |
| 9,259 | 860 | 8,399 | **Sistan and Baluchistan** |
| (100) | (9.29) | (90.71) |  |
| 10,253 | 1,300 | 8,953 | **South Khorasan** |
| (100) | (12.68) | )87.32( |  |
| 72,038 | 8,655 | 63,383 | **Tehran** |
| (100) | (12.01) | (87.99) |  |
| 20,621 | 2,908 | 17,713 | **West Azarbayjan** |
| (100) | (14.10) | (85.90) |  |
| 16,537 | 3,166 | 13,371 | **Yazd** |
| (100) | (19.14) | (80.86) |  |
| 8,249 | 868 | 7,381 | **Zanjan** |
| (100) | (10.52) | (89.48) |  |
| 468,947 | 63,637 | 405,310 | **Total** |
| (100) | (13.57) | (86.43) |  |

**3.4 The insulin consumption pattern**

Among the 38,947 pilgrims in 2022, 5,198 had diabetes. Among this number of diabetic patients, 7 types of insulin were injected. The highest type was related to Insulin Glargine (44.1%) and Insulin Aspart(41.3%) and the lowest Insulin Detemir (0.1%). Table 4 shows the results of this section

Table 4. Dose and number of insulin injections

| **Dosage** | **Drug Name** | **(%)** |
| --- | --- | --- |
| 100 IU/ml | Insulin (Regular) | (4.9) |
| 100 IU/ml | Insulin Aspart | (41.3) |
| 100 IU/ml | Insulin Biphasic Isophane | (5.6) |
| 100 IU/ml | Insulin Detemir | (0.1) |
| 300 IU/ml | Insulin Glargine | (44.1) |
| 100 IU/ml | Insulin Glulisin | (1.6) |
| 100 IU/ml | Insulin Isophane | (1.6) |
| **Total** | | **100** |

**3.5 DM Healthcare Delivery and Economic Burden:**

DM has been one of the most important risk factors and diseases among Hajj pilgrims. DM can raise morbidity and mortality and lead to an increase in the consumption of medical services and the economic burden of disease (Direct cost and indirect cost).

The trend of providing DM services to Hajj pilgrims was also reported in 2018, 2019, 2022 and 2023(Fig. 2). The highest rate of receiving DM services was in 2023 with 37160 and the lowest rate was in 2022 with 2727 cases requiring dialysis treatment. The results of our study show that according to the data from Hajj Pilgrimage Medical Centre, about 10% of the total medical services provided during the 2023 Hajj ceremony were for DM outpatient and inpatient services.

Number of DM healthcare services

|  | Number of HTN healthcare |
| --- | --- |
| 2018 | 3207 |
| 2019 | 3087 |
| 2022 | 2727 |
| 2023 | 37160 |

**Figure 2: Trend of DM Healthcare delivery**

**3.6 Factors associated with DM disease**

The impact of demographic variables and certain risk factors on DM has been evaluated. Given that the presence of diabetes is a dichotomous variable (DM=0 for absence, DM=1 for presence), a logistic regression model was employed. The data exhibited a clustered structure, suggesting that individuals within a province may share similarities in terms of certain environmental and genetic characteristics. As the objective of the study was not to draw inferences about the provinces, robust standard errors (Robust SE) were utilized to adjust for this clustering, thereby facilitating more accurate inferences (Table 5). This approach ensures a rigorous analysis while accounting for the inherent structure of the data.

Table 5. Results of logistic regression analysis for factors influencing the prevalence of DM

| DM | OR | Robust. St. Err. | | | t-value | p-value | [95% Conf | | Interval] | | Sig |
| --- | --- | --- | --- | --- | --- | --- | --- | --- | --- | --- | --- |
| Age | 1.039 | .003 | | | 13.83 | 0 | 1.034 | | 1.045 | | *** |
| Sex (Women=1) | 1.329 | .041 | | | 9.34 | 0 | 1.252 | | 1.411 | | *** |
| FBS ($\geq126$) | 29.842 | 1.407 | | | 72.02 | 0 | 27.208 | | 32.732 | | *** |
| Constant | .005 | .001 | | | -37.26 | 0 | .004 | | .006 | | *** |
|  | | | | | | | | | | | |
| Mean dependent var | | | 0.136 | SD dependent var | | | | 0.342 | |  |  |
| Pseudo r-squared | | | 0.303 | Number of obs | | | | 468947 | |  |  |
| Chi-square | | | 7623.526 | Prob > chi2 | | | | 0.000 | |  |  |
| Akaike crit. (AIC) | | | 259495.904 | Bayesian crit. | | | | 259540.137 | |  |  |
| **** p<.01, ** p<.05, * p<.1* | | | | | | | | | | | |

The chi-square statistic for the overall model was estimated to be 7623.526, indicating that the fitted model is statistically significant (p-value<0.001). The null hypothesis, which posits that all independent variables in the model do not affect the odds (probability) of having DM, has been rejected. This suggests that at least one of the independent variables in the model significantly influences the odds (probability) of developing DM. The p-value for all independent variables in the model was less than 0.05, implying that all variables significantly affect the odds of developing DM.

Our analysis reveals that, with each yearly increase in the age of the pilgrims (assuming other variables remain constant), the odds of having DM increase by 0.04. For the gender, the odds of having DM among women is 0.33 higher than among men, when the other variables are constant. Similarly, for the fasting blood sugar variable, the odds of having DM for individuals with a fasting blood sugar level greater or equal to 126 (FBS$\geq126$) are 29.84 times the odds of having DM for individuals with a fasting blood sugar level less than 126 (FBS$<126$), when other variables are considered to be constant.

4. **Discussion**

The primary objective of this study is to gather demographic data, estimate the prevalence of DM, and identify its influencing factors among Iranian pilgrims from 2012 to 2022. The analysis of DM prevalence in the target population reveals four key findings. The first finding shows that the highest prevalence of DM was observed in the age group of 70 years and above, followed by the group aged 45 to 70 years. A study conducted among individuals aged 35 to 70 years, using data from the Persian cohort study, estimated the age-standardized prevalence of diabetes at 15%, which aligns with the findings of the current research(10). A similar study on the trend of DM prevalence among individuals aged 25 to 64 years reported the prevalence of diabetes as 8.4%, 9%, 11.1%, and 13.2% in 2004, 2007, 2011, and 2016, respectively(11). The discrepancy between these findings and the current analysis could be attributed to the age differences of the study groups. A study conducted in Kerman province revealed that the overall prevalence of DM is 12%, ranging from 7.1% in the age group of 15-24 years to 18.4% in the age group of 55-64 years(12). The prevalence of DM in phase one was estimated at 10.8% and in phase two at 10.3%(12). These findings underscore the significant impact of age on the prevalence of DM among Iranian Hajj pilgrims.

The second finding of this study reveals that the prevalence of diabetes among women is higher than among men, with 12.51% of male pilgrims and 14.61% of female pilgrims diagnosed with this disease. A similar national-level study aligns with these findings, reporting the prevalence of DM as 13.5% in men, 14.7% in women, and 14.2% in both genders(13). A systematic review, which reported the prevalence of DM in 25 papers, found rates ranging from 1.6% among Omani pilgrims to 40% among Chinese pilgrims. The weighted cumulative prevalence of DM among all pilgrims was estimated at 5%. This review study reported the lowest rate of DM prevalence among Australian and Iranian pilgrims(14).. A study on the prevalence of chronic diseases in Iranian pilgrims referred to Iranian clinics located in Iraq found that 13.4% of the referred pilgrims suffer from diabetes, with a higher prevalence in female pilgrims than in males(15).. Another study assessing the health status of Iranian pilgrims before departing for Hajj over different years showed an increasing trend of diabetes prevalence from 2.44% in 2004 to 8.31% in 2008(16). A significant study on French pilgrims revealed that the rate of diabetes in French pilgrims is five times higher than that in the general population, with the prevalence of diabetes, hypertension, and obesity being higher in female pilgrims than in male pilgrims(17).. The prevalence of chronic diseases is higher in Arab and Muslim countries than in non-Muslim countries. Furthermore, a large portion of the participants in the Hajj are over 60 years old(18).. A study on chronic diseases of deceased hospitalized individuals in Saudi hospitals during the Hajj showed that one-third of the deceased individuals suffered from diabetes(19).

In 2015, it was estimated that there were 415 million people with diabetes between the ages of 20 to 79 years, 5 million deaths due to diabetes, and three-quarters of those with diabetes lived in low- and middle-income countries. It is predicted that the number of people with diabetes between the ages of 20 to 79 will reach 642 million by the year 2040 (20).. A study in Malaysia showed that 26.5% of Hajj pilgrims in that country suffer from hypertension and 15.4% from diabetes(21).. The findings of a review of 22 selected studies show that the main health problems of pilgrims during Hajj are pneumonia, influenza, and asthma, and diseases such as stroke and heart attack (16.67%) and diabetes (13.33%) (22). The study by Khan and colleagues shows that analgesics, antibiotics, antacids, antihistamines, as well as diabetes and blood pressure medications were the most commonly prescribed drugs for Indian pilgrims(8).. Evidence from a longitudinal study on South African pilgrims indicates that high blood pressure and mostly type 2 diabetes are the most common diseases in these individuals, with prevalence rates of 22.6% and 13.2% respectively(23).. A study on the risk factors for hospitalization and mortality among Indonesian pilgrims shows that diabetes significantly increases the risk of hospitalization in Hajj pilgrims(24).

The third finding of this study pertains to the variations in diabetes prevalence among pilgrims from different provinces. A study indicates that the lowest prevalence of diabetes is in Kermanshah province, while the highest rate is observed in Khuzestan province. This aligns with the findings of the current study, which also reports the highest rate of diabetes prevalence among pilgrims from Khuzestan province(13). However, a recently published study presents different findings, showing that the highest and lowest prevalence of diabetes are respectively in Yazd and Urmia. Another study reports that the highest prevalence of prediabetes was observed in Sari, while the lowest was in the city of Fasa(10). These findings underscore the significant regional disparities in diabetes prevalence among Hajj pilgrims.

The fourth finding of this study pertains to the risk factors and determinants of DM prevalence. The results of the current study indicate that a higher probability of developing DM is associated with increasing age, high fasting blood sugar in pilgrims, and female gender. A study in this field reveals that the prevalence of DM is higher in urban residents compared to rural ones and in individuals with lower education levels(13).. According to the findings of an internal study, age and gender are determinants of the 5-year incidence rate of diabetes, such that increasing age, high blood pressure, overweight, and obesity are associated with an increased risk of diabetes(25).

The results of multivariate logistic regression in the study by Johari and colleagues in Fars province (26) showed that the prevalence of DM is linked with increasing age, being single, family history of diabetes, abdominal obesity, and hypertension. An inverse statistical relationship was observed between the prevalence of DM and factors such as high triglycerides, living in rural areas, higher education, and employment(27). The significant increase in diabetes has occurred in all countries and both rural and urban areas. Accurate global, regional, and local estimates and predictions of DM prevalence are essential for planning and monitoring prevention and treatment strategies and assessing progress toward achieving the goals set globally for the control of non-communicable diseases and sustainable development.

In a prospective observational study, 26% of individuals hospitalized in the hospitals of the city of Mecca were diagnosed with diabetes, while previously they were unaware of their diabetes. Upon admission of patients, it was found that approximately 77% of the diabetic participants had poor diabetes control and about 55% had received diabetes education sessions before going to Hajj. The study findings showed that approximately 37% of hospitalized individuals sometimes monitor their blood sugar themselves and only 22% knew that self-monitoring of blood sugar during the Hajj is recommended(28).. These findings underscore the significant impact of demographic and health factors on the prevalence of DM among Hajj pilgrims.

Another finding of the study was related to the investigation of the dose and units of insulin consumption. Insulin should be used within a maximum of 28 days and its temperature should be between 4°C and 24°C. If insulin is not used, it should not be frozen or placed near the freezer. Also, for maximum effectiveness, it should be kept in a refrigerator at a temperature of about 4°C. Patients should pay attention to the dose insulin prescribed in the country of origin, as other countries may use different measurement tools. Many countries use a dose of 40 U/ml, but in Saudi Arabia, a dose of 100 U/ml is more common. Prior to travel, pilgrims should check with their health care provider for dosage requirements(29).

Some studies examined the challenges faced by diabetic pilgrims in ensuring the proper transportation and storage of insulin with the aim of maintaining its potency. Diabetic pilgrims should acquire adequate knowledge on how to store and store insulin during the travels. It is recommended that cool packs be used to maintain insulin at optimal temperature during transportation and storage. Pilgrims should also be adequately educated on insulin management strategies to minimize non-maintenance complications and ensure its quality(29, 30).

Hajj, one of the five pillars of Islam, is a mandatory religious duty for Muslims that must be performed at least once in their lifetime. Considering the global prevalence of diabetes at 8.8%, and the number of adult Muslims performing Hajj (approximately 2.5 million), it can be estimated that the number of Muslims with diabetes performing Hajj may exceed 220,000 annually. Islamic rulings stipulate that Hajj should not impose undue hardship on Muslims. The Holy Quran specifically exempts those who are physically or financially incapable from performing Hajj if it would result in harmful consequences for the individual. This exemption could apply to individuals with diabetes, given the severe and chronic complications associated with the condition. During Hajj, adherence to dietary guidelines, fluid intake, and mobility is crucial. Poor dietary control in pilgrims with diabetes can lead to serious complications and potentially fatal outcomes(7).

**5. Conclusion**

Accurate global, regional, and national estimates of diabetes prevalence are crucial for the planning and monitoring of prevention and treatment strategies, as well as for evaluating progress toward the goals set by joint global action plans for non-communicable disease control and sustainable development. Based on the studies, the present study is the most comprehensive to date, examining the prevalence of diabetes in Iranian pilgrims over the past decade, encompassing about half a million Iranian pilgrims. The results of the study reveal a significant variation in the prevalence of diabetes across different age groups, genders, and provinces. However, there appears to be a dearth of in-depth information on lifestyle adaptations and behavioral determinants among Hajj pilgrims concerning the management of chronic diseases during the Hajj. Therefore, appropriate screening, diagnosis, and management by primary care physicians are essential to prevent adverse health outcomes and mitigate the economic burden.

**Declaration of Competing Interest:**

The authors declare that they have no known competing financial interests or personal relationships that could have appeared to influence the work reported in this paper.

**Funding**: This research received no external funding.

**Consent Statement:** Not applicable.

**Acknowledgements:**

We appreciate the Hajj Pilgrimage Medical Centre, Iranian Red Crescent Society for offering the data used in our study. We also thank the respected reviewers who improved the quality of the article with their valuable comments. The data analyzed in this study can be accessed by sending a request to the corresponding author and the Hajj Pilgrimage Medical Centre, Iranian Red Crescent Society.

**Data Availability Statement:**

The datasets used and/or analyzed during the current study are available.

**CRediT authorship contribution statement**

**Pirhossein Kolivand:** Conceptualization, Project administration.

**Peyman Saberian:** Conceptualization, Writing – review & editing.

**Samad Azari:** Conceptualization, Data curation, Writing – review & editing.

**Fereshteh Karimi:** Data curation, Writing – original draft.

**Soheila Rajaei:** Data curation, Data analysis.

**Hossein Saffari:** Data curation, Writing – review & editing.

**Masoud Behzadifar:** Methodology, Visualization, Writing – review & editing.

**Arash Parvari:** Methodology, Visualization, formal analysis.

**Ali Marashi:** Conceptualization, Writing – review & editing, Investigation.

**Taher Doroudi:** Data curation, Conceptualization.

**Seyed Jafar Ehsanzadeh:** Writing – review & editing.

**Behzad Raei:** Writing – review & editing.

**References**

1.	Shirah BH, Al Nozha FA, Zafar SH, Kalumian HM. Mass gathering medicine (Hajj pilgrimage in Saudi Arabia): The outcome of cardiopulmonary resuscitation during Hajj. Journal of Epidemiology and Global Health. 2019;9(1):71.

2.	Freitag U. The History of the Hajj as Heritage: Asset or Burden to the Saudi State? Travelling Pasts: The Politics of Cultural Heritage in the Indian Ocean World: Brill; 2019. p. 62-79.

3.	Ong KL, Stafford LK, McLaughlin SA, Boyko EJ, Vollset SE, Smith AE, et al. Global, regional, and national burden of diabetes from 1990 to 2021, with projections of prevalence to 2050: a systematic analysis for the Global Burden of Disease Study 2021. The Lancet. 2023.

4.	Saeedi P, Petersohn I, Salpea P, Malanda B, Karuranga S, Unwin N, et al. Global and regional diabetes prevalence estimates for 2019 and projections for 2030 and 2045: Results from the International Diabetes Federation Diabetes Atlas. Diabetes research and clinical practice. 2019;157:107843.

5.	Atlas D. International diabetes federation. IDF Diabetes Atlas, 7th edn Brussels, Belgium: International Diabetes Federation. 2015;33(2).

6.	Shaikh S, Ashraf H, Shaikh K, Iraqi H, Ndour Mbaye M, Kake A, et al. Diabetes Care During Hajj. Diabetes Therapy. 2020;11:2829-44.

7.	Ibrahim M, Abdelaziz SI, Almagd MA, Alarouj M, Annabi FA, Armstrong DG, et al. Recommendations for management of diabetes and its complications during Hajj (Muslim pilgrimage). BMJ Open Diabetes Research & Care. 2018;6(1).

8.	Khan ID, Khan SA, Asima B, Hussaini SB, Zakiuddin M, Faisal F. Morbidity and mortality amongst Indian Hajj pilgrims: A 3-year experience of Indian Hajj medical mission in mass-gathering medicine. Journal of infection and public health. 2018;11(2):165-70.

9.	Memish ZA, Stephens GM, Steffen R, Ahmed QA. Emergence of medicine for mass gatherings: lessons from the Hajj. The Lancet infectious diseases. 2012;12(1):56-65.

10.	Khamseh ME, Sepanlou SG, Hashemi-Madani N, Joukar F, Mehrparvar AH, Faramarzi E, et al. Nationwide prevalence of diabetes and prediabetes and associated risk factors among Iranian adults: analysis of data from PERSIAN cohort study. Diabetes Therapy. 2021;12:2921-38.

11.	Khodakarami R, Abdi Z, Ahmadnezhad E, Sheidaei A, Asadi-Lari M. Prevalence, awareness, treatment and control of diabetes among Iranian population: results of four national cross-sectional STEPwise approach to surveillance surveys. BMC Public Health. 2022;22(1):1216.

12.	Najafipour H, Farjami M, Sanjari M, Amirzadeh R, Shadkam Farokhi M, Mirzazadeh A. Prevalence and incidence rate of diabetes, pre-diabetes, uncontrolled diabetes, and their predictors in the adult population in southeastern Iran: findings from KERCADR study. Frontiers in public health. 2021;9:611652.

13.	Azadnajafabad S, Ahmadi N, Rezaei N, Rashidi M-M, Saeedi Moghaddam S, Mohammadi E, et al. Evaluation of the diabetes care cascade and compliance with WHO global coverage targets in Iran based on STEPS survey 2021. Scientific Reports. 2023;13(1):13528.

14.	Yezli S, Mushi A, Almuzaini Y, Balkhi B, Yassin Y, Khan A. Prevalence of diabetes and hypertension among Hajj pilgrims: a systematic review. International journal of environmental research and public health. 2021;18(3):1155.

15.	Mousavi J, Jafari F. Prevalence of diseases in pilgrims referring to Iranian clinics in Iraq. International Journal of Travel Medicine and Global Health. 2016;4(1):31-3.

16.	Razavi SM, Sabouri-Kashani A, Ziaee-Ardakani H, Tabatabaei A, Karbakhsh M, Sadeghipour H, et al. Trend of diseases among Iranian pilgrims during five consecutive years based on a Syndromic Surveillance System in Hajj. Medical journal of the Islamic Republic of Iran. 2013;27(4):179.

17.	Gautret P, Bauge M, Simon F, Benkouiten S, Valéro R, Parola P, et al. Overweight and obesity in French Hajj pilgrims. Journal of immigrant and minority health. 2013;15:215-8.

18.	Roglic G. WHO Global report on diabetes: A summary. International Journal of Noncommunicable Diseases. 2016;1(1):3-8.

19.	Gaddoury MA, Armenian HK. Epidemiology of hajj pilgrimage mortality: Analysis for potential intervention. Journal of Infection and Public Health. 2023.

20.	Ogurtsova K, da Rocha Fernandes J, Huang Y, Linnenkamp U, Guariguata L, Cho NH, et al. IDF Diabetes Atlas: Global estimates for the prevalence of diabetes for 2015 and 2040. Diabetes research and clinical practice. 2017;128:40-50.

21.	Hashim S, Ayub ZN, Mohamed Z, Hasan H, Harun A, Ismail N, et al. The prevalence and preventive measures of the respiratory illness among Malaysian pilgrims in 2013 Hajj season. Journal of travel medicine. 2016;23(2):tav019.

22.	Al-Masud SMR, Bakar AA, Yussof S. Determining the types of diseases and emergency issues in Pilgrims during Hajj: A literature review. Stat Inf. 2016;5(7).

23.	Mushi A, Yassin Y, Khan A, Alotaibi B, Parker S, Mahomed O, et al. A longitudinal study regarding the health profile of the 2017 South African Hajj pilgrims. International Journal of Environmental Research and Public Health. 2021;18(7):3607.

24.	Ardiana M, Paramitha AD, Shabrina FA, Harsoyo PM. Risk Factors of Hospitalization and Mortality among Hajj Pilgrims in Indonesia. 2023.

25.	Ebrahimi H, Emamian MH, Hashemi H, Fotouhi A. High incidence of diabetes mellitus among a middle-aged population in Iran: a longitudinal study. Canadian journal of diabetes. 2016;40(6):570-5.

26.	Iranmanesh M, Moghavvemi S, Zailani S, Hyun SS. The role of trust and religious commitment in Islamic medical tourism. Asia Pacific Journal of Tourism Research. 2018;23(3):245-59.

27.	Johari MG, Jokari K, Mirahmadizadeh A, Seif M, Rezaianzadeh A. The prevalence and predictors of pre-diabetes and diabetes among adults 40–70 years in Kharameh cohort study: A population-based study in Fars province, south of Iran. Journal of Diabetes & Metabolic Disorders. 2022:1-11.

28.	Hasan G, Moabber H, Alyamani A, Sayeed A, Altatar F. Study on risk factors (predisposing factors) for poor diabetes control during Hajj (1436/2015) in people with diabetes. Pakistan journal of medical sciences. 2016;32(5):1092.

29.	Algeffari M. Diabetes and Hajj pilgrims: A Narrative review of literature. JPMA The Journal of the Pakistan Medical Association. 2019;69(6):879-84.

30.	Yezli S, Yassin Y, Mushi A, Balkhi B, Khan A. Insulin knowledge, handling, and storage among diabetic pilgrims during the Hajj Mass Gathering. Journal of Diabetes Research. 2021;2021.
